# A multi-layered poroelastic slab model under cyclic loading for a single osteon

**DOI:** 10.1186/s12938-018-0528-y

**Published:** 2018-07-17

**Authors:** Yaogeng Chen, Wenshuai Wang, Shenghu Ding, Xu Wang, Qun Chen, Xing Li

**Affiliations:** 1 School of Mathematics and Statistics, Ningxia University, Helanshan Road 489, Yinchuan, 750021 Ningxia Province People’s Republic of China; 2 School of Science, Ningxia Medical University, Yinchuan, 750004 China

**Keywords:** Osteon, Interstitial fluid flow, Cyclic loading, Lamellar structure, Osteocytes

## Abstract

**Background:**

An osteon consists of a multi-layered bone matrix and interstitial fluid flow in the lacunar–canalicular system. Loading-induced interstitial fluid flow in the lacunar–canalicular system is critical for osteocyte mechanotransduction and bone remodelling.

**Methods:**

To investigate the effects of the lamellar structure and heterogeneous material properties of the osteon on the distributions of interstitial fluid flow and seepage velocity, an osteon is idealized as a hollow two-dimensional poroelastic multi-layered slab model subjected to cyclic loading. Based on poroelastic theory, the analytical solutions of interstitial fluid pressure and seepage velocity in lacunar–canalicular pores were obtained.

**Results:**

The results show that strain magnitude has a greater influence on interstitial fluid pressure than loading frequency. Interestingly, the heterogeneous distribution of permeability produces remarkable variations in interstitial fluid pressure and seepage velocity in the cross-section of cortical bone. In addition, interstitial fluid flow stimuli to osteocytes are mostly controlled by the value of permeability at the surface of the osteon rather than at the inner wall of the osteon.

**Conclusion:**

Interstitial fluid flow induced by cycling loading stimuli to an osteocyte housed in a lacunar–canalicular pore is not only correlated with strain amplitude and loading frequency, but also closely correlated with the spatial gradient distribution of permeability. This model can help us better understand the fluid flow stimuli to osteocytes during bone remodelling.

## Background

Bone is a poroelastic material often subject to physiological cyclic loadings that arise from walking, running, or other daily activities [1, 2]. These activities can cause the interstitial fluid flow in bone, which is believed to initiate a mechanical response in osteocytes for bone remodelling [2–6]. Thus, linking bone cyclic loading to local cortical bone tissue remodelling is an issue of great interest to understand the rate of bone tissue renewal [7].

**Fig. 1:**
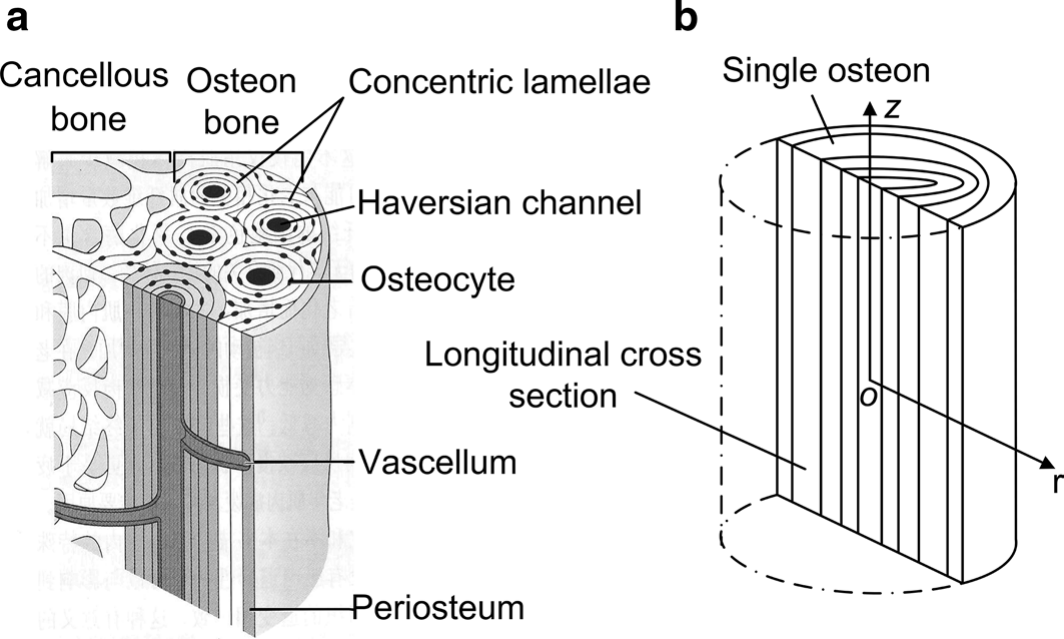
**a** Microstructure of bone. **b** Longitudinal cross section of a single cylindrical osteon

Cortical bone makes up of osteons in interstitial bone tissue. The osteon is a fundamental building unit of the cortical bone at the microscopic scale (Fig. 1a), which consists of roughly multi-layered cylindrical composites of mineralized fibres arranged in 3–5 μm thick lamellae around a central Haversian system (Fig. 1b), and each lamella has different mechanical properties [7]. Beno et al. [8] pointed out that the lacunar–canalicular permeability coefficients vary in three orders of magnitude. Although the role of properties such as permeability in fluid flows inside the osteon is widely studied, the effect of spatial gradient distribution of properties on mechanical stimuli during bone remodeling remains unclear.

Since experimental work at the osteon scale does not seem feasible at the moment, various theoretical models based on Biot’s poroelastic theory have been used to quantitatively evaluate the strain-induced interstitial fluid in cortical bone. Some of the analytical models mentioned above consider the bone tissue as a transverse isotropic poroelastic material [1, 7, 9]. One of the studies is the work of Rémond and Naili [9], who modelled the osteon as a hollow cylinder under cyclic loading and obtained analytical solutions of pressure distribution and mass flux to investigate bone remodelling. This work was extended by Wu et al. [1] to demonstrate the loading conditions and material parameters on the distribution of fluid flow. Meanwhile, computational approaches are essential to the elucidation of the mechanical stimuli to osteocytes for bone remodelling. A finite element poroelastic model was developed to investigate the effect of the spatial gradients of material properties on interstitial bone fluid pressure, and showed that permeability variations have no significant consequence on radial fluid velocity [7]. Animal-specific finite element models were presented combining micro-CT reconstructions of the bone microstructure, to investigate whether microstructural changes associated with osteoporosis can affect the interstitial fluid flow around osteocytes [10]. In the above studies, most studies treated the single osteon as a homogeneous poroelastic material, yet the behaviour of the material is very sensitive to the spatial variation of properties such as the permeability parameter within the constituents. Therefore, the effects of material heterogeneity between each lamella on the interstitial fluid flow that stimulates osteocytes to remodel bone are not completely understood.

The purpose of this study was thus twofold: firstly, to develop a multi-layered poroelastic slab model composed of multiple layers to quantify parameter values for the osteon structures. Secondly, to apply the model with calculated parameter values to investigate the specific influence of spatial gradient distribution of lacunar–canalicular permeability on fluid pressure and velocity in the lacunar–canalicular system.

## Methods

### Description of the geometry

Biot’s poroelasticity theory is used to account for the fluid–solid interactions in this multilayer model of an osteon. After giving the description of the geometry is given and the fundamental equations of poroelasticity are stated, the boundary and initial conditions corresponding to the osteon model are specified.

**Fig. 2:**
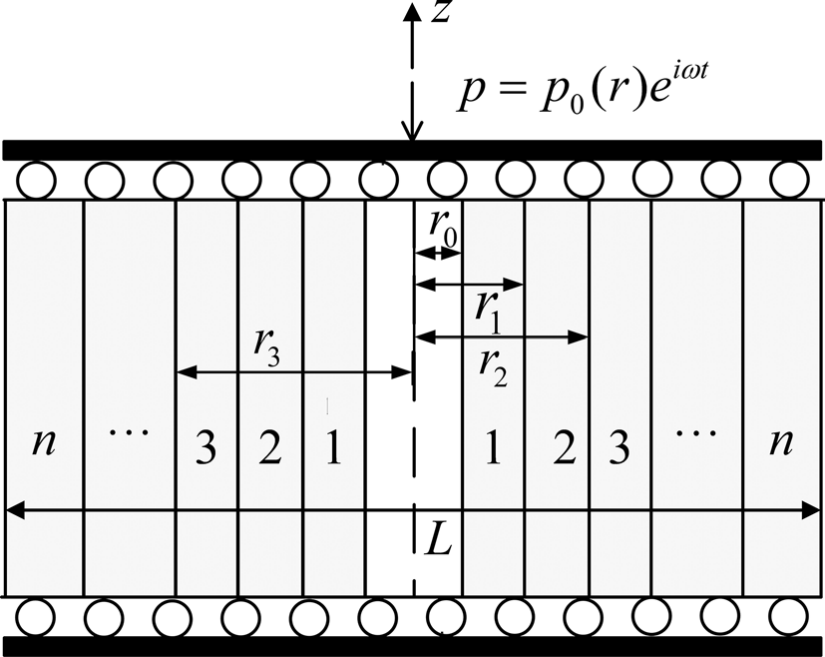
The single osteon model is shaped as a two-dimensional hollow poroelastic slab consisting of *n* layers with cyclic loading applied in the longitudinal direction

As illustrated in Fig. 2, a single osteon is idealized as a two-dimensional poroelastic hollow slab composed of multiple layers with a width of *L* in the *r*-direction, where each layer is assumed to be a transverse isotropic material and has a different value of permeability. The model represents the longitudinal cross section of a single cylindrical osteon. Here, we neglected the Haversian canal at the centre of the osteon. The matrix material and fluid are assumed to be compressible. We assumed that fluid can flow freely from the outer and the inner boundaries of the osteon. The model is supposed to be axisymmetric and the symmetry axis of the material is defined as *z*, so that the interstitial fluid flow can flow only in the radial direction. Physical quantities associated with the *i*-th layer are recognized by the subscript *i*. Additionally, all lamellae are assumed to be perfectly bonded between them. To apply axial strain loading on the osteon, two rigid and impermeable plates are placed at the top and bottom of this model.

### Governing equations

Considering the problem symmetry, the stress components are $\sigma_{\theta r} = \sigma_{\theta z} = \sigma_{zr} = 0$, and the displacement components is $u_{\theta } = 0$, and therefore the constitutive laws for the osteon in a low-frequency cyclic loading such as walking can be written as [11, 12]:

1 $$ \sigma_{rr} = M_{11} \varepsilon_{rr} + M_{12} \varepsilon_{\theta \theta } + M_{13} \varepsilon_{zz} - \alpha p, $$

2 $$ \sigma_{\theta \theta } = M_{12} \varepsilon_{rr} + M_{11} \varepsilon_{\theta \theta } + M_{13} \varepsilon_{zz} - \alpha p. $$

where, $\sigma_{ij} , \, \varepsilon_{ij}$ and $p$ are, respectively, the total stress and strain components and the interstitial fluid pressure in the cylindrical coordinate system; $\alpha$ is Biot’s effective stress coefficients in the isotropic plane (*r*-$\theta$ plane); and the drained elasticity tensor $M_{ij}$ of the solid skeleton can be expressed in the following form [11, 12]:

3 $$ \begin{aligned} M_{11} &= E_{r} (E_{z} - E_{r} \mu_{z}^{2} )(1 + \mu_{r} )^{ - 1} (E_{z} - E_{z} \mu_{r} - 2E_{r} \mu_{z}^{2} )^{ - 1} , \hfill \\ M_{12} &= E_{r} (E_{z} \mu_{r} - E_{r} \mu_{z}^{2} )(1 + \mu_{r} )^{ - 1} (E_{z} - E_{z} \mu_{r} - 2E_{r} \mu_{z}^{2} )^{ - 1} , \hfill \\ M_{13} & = E_{r} E_{z} \mu_{z} (E_{z} - E_{z} \mu_{r} - 2E_{r} \mu_{z}^{2} )^{ - 1} , \hfill \\ \end{aligned} $$

in which $E_{r}$ and $\mu_{r}$ are drained Young’s modulus and Poisson’s ratio in the isotropic plane, respectively, and $E_{z}$ and $\mu_{z}$ are similar quantities related to the direction of the axis of symmetry.

Continuity equation is also known as liquid mass conservation equation. The model in this paper based on cylindrical geometry of the osteon and its poroelastic properties. Material is modeled as transverse isotropic. Both fluid and solid phases are supposed to be compressible. Therefore, the flow of interstitial fluid in osteon to satisfy the continuity equation as follows [12]:

4 $$ \frac{\partial \xi }{\partial t} = \frac{k}{\mu }\nabla^{2} p, $$

where $k$ is the intrinsic permeability in the isotropic plane and $\mu$ is the viscosity of the pore fluid, and $\nabla^{2}$ is a differential operator.

The fluid volumetric variation relation for a transversely isotropic material may be written as:

5 $$ p = N\left[ {\xi - \left( {\alpha \varepsilon_{rr} + \alpha \varepsilon_{\theta \theta } + \alpha^{\prime}\varepsilon_{zz} } \right)} \right], $$

where $\xi$ is the variation of fluid content per unit reference volume. $N$ is Biot’s modulus and $\alpha^{\prime}$ is Biot’s effective stress coefficients in the *z*-direction.

Usually, bone is subjected to low-frequency cyclic loading from the activities of daily life. The equilibrium equations and the strain displacement relation can be written as follows:

6 $$ \frac{{\partial \sigma_{rr} }}{\partial r} + \frac{{\sigma_{rr} - \sigma_{\theta \theta } }}{r} = 0, $$

7 $$ \varepsilon_{rr} = \frac{{\partial u_{r} }}{\partial r}, $$

8 $$ \varepsilon_{\theta \theta } = \frac{{u_{r} }}{r}, $$

where $u_{r}$ is the components of the vector of displacement in the cylindrical coordinate system.

Substituting Eqs. (1) and (2) into equilibrium equation Eq. (6), we obtain:

9 $$ \frac{\partial }{\partial r}\left( {M_{11} \varepsilon_{rr} + M_{12} \varepsilon_{\theta \theta } } \right) + \frac{{\left( {M_{11} - M_{12} } \right)\left( {\varepsilon_{rr} - \varepsilon_{\theta \theta } } \right)}}{r} - \alpha \frac{\partial p}{\partial r} = 0. $$

Then, substituting Eqs. (7) and (8) into (9), we obtain:

10 $$ M_{11} \frac{\partial }{\partial r}\left( {\frac{{\partial u_{r} }}{\partial r} + \frac{{u_{r} }}{r}} \right) - \alpha \frac{\partial p}{\partial r} = 0. $$

Considering the problem symmetry and fluid flow only in the radial direction, the radial displacement $u_{i,r}$ and fluid pressure *p* depend only on *r* and *t*. We assume that the axial cyclic loading is in the form of an applied strain [2], $\varepsilon_{z} (t) = \varepsilon_{z0} e^{i\omega t}$, where $\varepsilon_{z0}$ and $\omega$ are the amplitude of the cyclic strain and its angular frequency, respectively. Therefore, the stress components, the vector of displacement and the interstitial fluid pressure in each layer have the solution forms of $\sigma_{rr} = \sigma_{i,r0} (r)e^{i\omega t} ,$ $\sigma_{\theta \theta } = \sigma_{i,\theta 0} (r)e^{i\omega t}$, $u_{i,r} = u_{i,r0} (r)e^{i\omega t}$ and $p = p_{i,0} e^{i\omega t}$ [7, 9, 13], where $\sigma_{i,r0}$, $\sigma_{i,\theta 0}$, $u_{i,r0}$ and $p_{i,0}$ are the radial stress amplitude, the radial displacement amplitude and the interstitial fluid pressure amplitude in each layer of the lamellar bone. Thus, Eq. (10) can be expressed by:

11 $$ \frac{{du_{i,r0} (r)}}{dr} + \frac{{u_{i,r0} (r)}}{r} = \frac{{\alpha p_{i,o} (r)}}{{M_{11} }} + c_{i} , $$

where $c_{i}$ is the integral constant determined by the boundary conditions.

Substituting Eqs. (7) and (8) into equilibrium equation Eq. (2), we obtain:

12 $$ \xi = \alpha \frac{{\partial u_{r} }}{\partial r} + \alpha \frac{{u_{r} }}{r} + \alpha^{\prime}\varepsilon_{z0} e^{i\omega t} + p/N, $$

Substituting Eq. (12) into the continuity Eq. (4), we obtain:

13 $$ \frac{\partial }{\partial t}\left( {\alpha \frac{{\partial u_{r} }}{\partial r} + \alpha \frac{{u_{r} }}{r} + \alpha^{\prime}\varepsilon_{z0} e^{i\omega t} + p/N} \right) - \frac{k}{\mu }\nabla^{2} p = 0. $$

Applying $u_{i,r} = u_{i,r0} (r)e^{i\omega t} ,{\kern 1pt} {\kern 1pt} p = p_{i,0} e^{i\omega t}$ and substituting Eq. (11) into Eq. (13) leads to the differential equation of $p_{i,0} (r)$ in each bone lamella to be derived as:

14 $$ \frac{{d^{2} p_{i,0} (r)}}{{dr^{2} }} + \frac{1}{r}\frac{{dp_{i,0} (r)}}{dr} - \frac{{i\omega \mu (M_{11} + N\alpha^{2} )}}{{kMM_{11} }}p_{i,0} (r) = \frac{i\omega \mu }{k}(\alpha c + \alpha^{'} \varepsilon_{z0} ). $$

### Interface and boundary conditions

We considered normal physiological activities to have low frequencies of loading. The Haversian canal in the centre of the osteon plays a reservoir role. The interstitial fluid pressure, fluid flux, displacement and stress are considered continuous at the boundaries of the layer. Therefore, the initial and boundary conditions for interstitial fluid pressure *p* can be described by:

1. The osteon is in a state of balance before cyclic loading is applied, and the fluid pressure in each layer of the osteon is null:
  15 $$ t = 0;\;p_{i,0} = 0\;(i = 1, \cdots ,n), $$
2. At both end of the osteon ($r = r_{0} = a$ and $r = r_{n} = b$), the fluid flow pressure is assumed to be null. This boundary condition means that the cement surface of the osteon is supposed to be full permeable. It is significant to use this permeable case for stimulating the presence of the micro-cracks.
  16 $$ p_{1,0} = p_{n,0} = 0; $$
3. At the interface between two successive layers of the osteon ($r = r_{i},\;\,i = 1,2, \ldots ,n - 1$), the fluid pressure, displacement, normal velocity and normal stress are continuous:
  17 $$ p_{i,0} = p_{i + 1,0} ,\quad - \frac{{\kappa_{i} }}{\mu }\frac{{\partial p_{i,0} }}{{\partial r_{i} }} = - \frac{{\kappa_{i + 1} }}{\mu }\frac{{\partial p_{i + 1,0} }}{{\partial r_{i + 1} }}, $$
  18 $$ u_{i,r0} = u_{i + 1,r0} ,\quad \sigma_{i,r0} = \sigma_{i + 1,r0} , $$

### Solution for interstitial fluid pressure and seepage velocity

The fundamental solution for interstitial fluid pressure can be obtained by solving the differential Eq. (14) as follows:

19 $$ p_{i,0} (r) = - \frac{{NM_{11} (\alpha c_{i} + \alpha^{\prime}\varepsilon_{z0} )}}{{M_{11} + N\alpha^{2} }} + A_{i} I_{0} (Cr) + B_{i} K_{0} (Cr), $$

where $A_{i}$, $B_{i}$ and $c_{i}$ are unknown coefficients to be determined by the boundary conditions, and $I_{0}$ and $K_{0}$ denote the first kind and the second kind modified Bessel function of order zero, respectively. By substituting Eqs. (15)–(17) into Eq. (19), these equations can be written as follows in matrix form:

20 $$ [a_{kl} ]\left\{ {\begin{array}{*{20}c} {A_{1} } \\ {B_{1} } \\ \vdots \\ {A_{n} } \\ {B_{n} } \\ \end{array} } \right\} = \left\{ {\begin{array}{*{20}c} {f_{1} } \\ 0 \\ \vdots \\ 0 \\ {f_{2n} } \\ \end{array} } \right\},\;(k,l = 1, \ldots ,2n), $$

where the nonzero elements between $a_{kl}$ and $f_{k}$ are given by:

21 $$ \begin{aligned} a_{11} & = I_{0} (C_{ 1} a),\quad a_{12} = K_{0} (C_{ 1} a), \\ a_{2i,2i - 1} & = I_{0} (C_{i} r_{i} ),\quad a_{2i,2i} = K_{0} (C_{i} r_{i} ),\quad a_{2i,2i + 1} = - I_{0} (C_{i} r_{i} ),\quad a_{2i,2i + 2} = - K_{0} (C_{i} r_{i} ), \\ a_{2i + 1,2i - 1} & = \kappa_{i} I_{1} (C_{i} r_{i} ),\quad a_{2i + 1,2i} = - \kappa_{i} K_{1} (C_{i} r_{i} ),\quad a_{2i + 1,2i + 1} = - \kappa_{i + 1} I_{1} (C_{i + 1} r_{i} ), \\ a_{2i + 1,2i + 2} & = \kappa_{i + 1} K_{1} (C_{i + 1} r_{i} ),\quad (i = 1, \ldots ,n - 1), \\ a_{2n,2n - 1} & = I_{0} (C_{n} b),\quad a_{2n,2n} = K_{0} (C_{n} b), \\ f_{1} & = \frac{{NM_{11} (\alpha c_{1} + \alpha^{\prime}\varepsilon_{z0} )}}{{M_{11} + N\alpha^{2} }},\quad f_{2i} = \frac{{NM_{11} (\alpha c_{i} + \alpha^{\prime}\varepsilon_{z0} )}}{{M_{11} + N\alpha^{2} }} - \frac{{NM_{11} (\alpha c_{i + 1} + \alpha^{\prime}\varepsilon_{z0} )}}{{M_{11} + N\alpha^{2} }}, \\ f_{2i + 1} & = 0,\quad f_{2n} = - \frac{{NM_{11} (\alpha c_{n} + \alpha^{\prime}\varepsilon_{z0} )}}{{M_{11} + N\alpha^{2} }}. \\ \end{aligned} $$

By applying Cramer’s rule to Eq. (21), we can obtain the interstitial fluid pressure solution with the unknown coefficient $c_{i}$.

According the governing Eqs. (2)–(7), we can also obtain the differential equation related to the displacement of each bone lamella by:

22 $$ \frac{{\partial u_{i,r0} (r)}}{\partial r} + \frac{{u_{i,r0} (r)}}{\partial r} = \left[ { - \frac{{N\alpha (\alpha c_{i} + \alpha^{\prime}\varepsilon_{z0} )}}{{M_{11} + M\alpha^{2} }} + c_{i} } \right] + \frac{{\alpha A_{i} }}{{M_{11} }}I_{0} (Cr) + \frac{{\alpha B_{i} }}{{M_{11} }}K_{0} (Cr). $$

The fundamental solution for Eq. (22) can be written as

23 $$ u_{i,r0} = \frac{1}{2}r\left[ { - \frac{{N\alpha (\alpha c_{i} + \alpha^{\prime}\varepsilon_{z0} )}}{{M_{11} + M\alpha^{2} }} + c_{i} } \right] + \frac{\alpha }{{CM_{11} }}\left[ {A_{i} I_{1} (Cr) - B_{i} K_{1} (Cr)} \right] + \frac{{s_{i} }}{r}, $$

where $I_{ 1}$ and $K_{ 1}$ are the modified Bessel function of the first order.

Using the boundary condition (18), the following equations can be derived:

24 $$ \begin{aligned} &\frac{1}{2}r_{i} \left[ { - \frac{{N\alpha (\alpha c_{i} + \alpha^{\prime}\varepsilon_{z0} )}}{{M_{11} + N\alpha^{2} }} + c_{i} } \right] + \frac{\alpha }{{CM_{11} }}\left[ {A_{i} I_{1} (Cr_{i} ) - B_{i} K_{1} (Cr_{i} )} \right] + \frac{{s_{i} }}{{r_{i} }} \hfill \\ &= \frac{1}{2}r_{i} \left[ { - \frac{{N\alpha (\alpha c_{i + 1} + \alpha^{\prime}\varepsilon_{z0} )}}{{M_{11} + N\alpha^{2} }} + c_{i + 1} } \right] + \frac{\alpha }{{CM_{11} }}\left[ {A_{i + 1} I_{1} (Cr_{i} ) - B_{i + 1} K_{1} (Cr_{i} )} \right] + \frac{{s_{i + 1} }}{{r_{i} }}, \hfill \\ &\frac{1}{2}M_{11} \left[ { - \frac{{N\alpha (\alpha c_{i} + \alpha^{\prime}\varepsilon_{z0} )}}{{M_{11} + N\alpha^{2} }} + c_{i} } \right]{ + }\frac{\alpha }{C} \times \left\{ {C[A_{i} I_{0} (Cr_{i} ) + B_{i} K_{0} (Cr_{i} )] - \frac{1}{r}[A_{i} I_{1} (Cr_{i} ) - B_{i} K_{1} (Cr_{i} )]} \right\} \hfill \\ & {\kern 1pt}  - M_{11} \frac{{s_{i} }}{{r_{i}^{2} }} + \frac{1}{2}M_{12} \left[ { - \frac{{N\alpha (\alpha c_{i} + \alpha^{\prime}\varepsilon_{z0} )}}{{M_{11} + N\alpha^{2} }} + c_{i} } \right] + \frac{{\alpha M_{12} }}{{l_{i} CM_{11} }}[A_{i} I_{1} (Cr_{i} ) - B_{i} K_{1} (Cr_{i} )] + M_{12} \frac{{s_{i} }}{{r_{i}^{2} }} \hfill \\ &= \frac{1}{2}M_{11} \left[ { - \frac{{N\alpha (\alpha c_{i + 1} + \alpha^{\prime}\varepsilon_{z0} )}}{{M_{11} + N\alpha^{2} }} + c_{i + 1} } \right] + \frac{\alpha }{C} \times \left\{ {C[A_{i + 1} I_{0} (Cr_{i} ) + B_{i + 1} K_{0} (Cr_{i} )] - \frac{1}{r}[A_{i + 1} I_{1} (Cr_{i + 1} ) - B_{i + 1} K_{1} (Cr_{i + 1} )]} \right\} \hfill \\ & \quad - M_{11} \frac{{s_{i + 1} }}{{r_{i}^{2} }} + \frac{1}{2}M_{12} \left[ { - \frac{{N\alpha (\alpha c_{i + 1} + \alpha^{\prime}\varepsilon_{z0} )}}{{M_{11} + N\alpha^{2} }} + c_{i + 1} } \right] + \frac{{M_{12} \alpha }}{{r_{i} CM_{11} }}[A_{i + 1} I_{1} (Cr_{i} ) - B_{i + 1} K_{1} (Cr_{i} )] + M_{12} \frac{{s_{i + 1} }}{{r_{i}^{2} }}. \hfill \\ \end{aligned} $$

From the above linear equations, the unknown coefficient $c_{i}$ can be obtained. Substituting $A_{i,} \;B_{i,} \;c_{i}$ into Eq. (19), we get the analytical solution for the interstitial fluid pressure on each bone lamella.

To understand the mechanism of mechanical stimuli given to interstitial fluid flow, we investigated the seepage velocity in the lacunar–canalicular pores According to Darcy’s law [9], the seepage velocity $q_{r}$ for the *i*th layer is derived as follows:

25 $$ q_{r} = - \frac{k}{\mu }\frac{{\partial p_{i,0} (r)}}{\partial r} $$

### Numerical parameters

For the purpose of investigating the effect of spatial gradients of permeability on interstitial fluid flow and seepage velocity, Eqs. (19) and (25) are used for the parametric studies. The geometric and transverse isotropic poroelastic material properties for an osteon are listed in Table 1 [7, 11, 13–16]. The range of strain is selected between 0.04 and 0.3% to study the poroelastic response of a loaded osteon bone [17]. The loading frequency is chosen to be 1–21 Hz, which correspond to those of physiological activities [3]. The geometry of the inner and outer radii of the osteon is defined as: *a *= 50 μm and *b *= 150 μm [13], respectively. Here, we assume that a single osteon is composed of six layers, with each of the concentric lamellae having the same width.

**Table 1:** Geometrical and material properties of cortical bone as poroelastic material [7, 11, 13–16]

Symbol (unit)	Description	Value
$E_{r}$ (GPa)	Drained Young’s modulus in the isotropic plane (*r*-$\theta$ plane)	15.9
$E_{z}$ (GPa)	Drained Young’s modulus in the *z*-direction	20.3
$M$ (GPa)	Biot’s modulus	38
$\mu_{r}$	Poisson’s ratio in the isotropic plane	0.328
$\mu_{z}$	Poisson’s ratio in the *z*-direction	0.25
$\alpha$	Biot’s effective stress coefficients in the isotropic plane (*r*-$\theta$ plane)	0.132
$\alpha^{\prime}$	Biot’s effective stress coefficients in the *z*-direction	0.092
$\mu$ (*Pa* *s*)	Viscosity of the pore fluid	10^−3^
$a$ (μm)	Inner radius of the osteon	50
$b$ (μm)	Outer radius of the osteon	150

Among the material properties listed in Table 1, the permeability of each lamella is extremely difficult to determine due to the heterogeneity of the bone material and the multiscale structure of porous bone [4, 18–20]. According to previous theoretical and experimental results, the lacuna-canalicular permeability of cortical bone exhibits a broad variability, with values ranging from 10^−17^ to 10^−25^ m^2^ [18]. In our study, we chose the low value of 10^−18^ m^2^ for the lacunar–canalicular permeability (PLC) as a reference case to investigate lacunar–canalicular permeability effects on the behaviour of interstitial fluid flow. Here, we considered three cases listed in Table 2. In Case 1, permeability is linearly distributed from the first layer to the last layer. In both Case 2 and Case 3, lacunar–canalicular permeability has a symmetric distribution about the central axis ($r = L/2$). Around the centre of cortical bone, interstitial fluid in Case 2 can move more rapidly around the centre of the osteon than fluid close to the osteon surfaces, while Case 3 represents the reverse condition.

**Table 2:** Settings of single cortical bone permeability for poroelastic analysis

Permeability	$k_{1}$	$k_{2}$	$k_{3}$	$k_{4}$	$k_{5}$	$k_{6}$
Case 1	$0.5 \times 1 0^{ - 1 8}$	$0. 7\times 1 0^{ - 1 8}$	$0. 9\times 1 0^{ - 1 8}$	$1. 1\times 1 0^{ - 1 8}$	$1. 3\times 1 0^{ - 1 8}$	$1. 5\times 1 0^{ - 1 8}$
Case 2	$0.5 \times 1 0^{ - 1 8}$	$1. 0\times 1 0^{ - 1 8}$	$1.5 \times 1 0^{ - 1 8}$	$1.5 \times 1 0^{ - 1 8}$	$1. 0\times 1 0^{ - 1 8}$	$0.5 \times 1 0^{ - 1 8}$
Case 3	$1.5 \times 1 0^{ - 1 8}$	$1. 0\times 1 0^{ - 1 8}$	$0.5 \times 1 0^{ - 1 8}$	$0.5 \times 1 0^{ - 1 8}$	$1. 0\times 1 0^{ - 1 8}$	$1.5 \times 1 0^{ - 1 8}$

## Results

### Effects of stain amplitude and loading frequency on fluid flow pressure

**Fig. 3:**
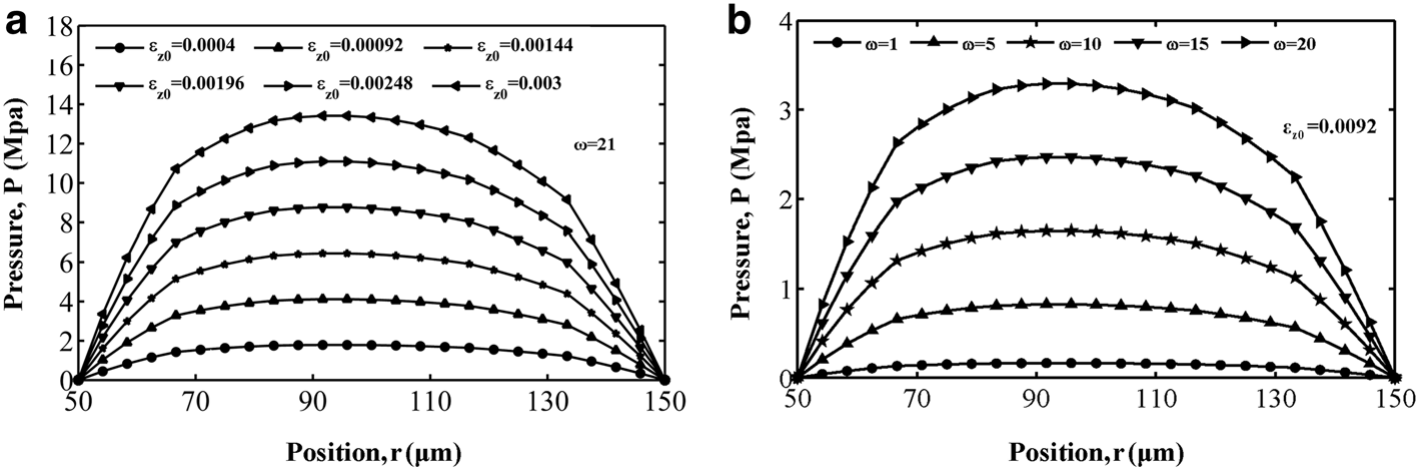
Fluid pressure distribution along the radial displacement *r* in case 1: **a** different strain amplitude at frequency of loading $\omega = 21$ and **b** different loading frequency at strain amplitude $\varepsilon_{z0} = 0. 0 0 0 9 2$

**Fig. 4:**
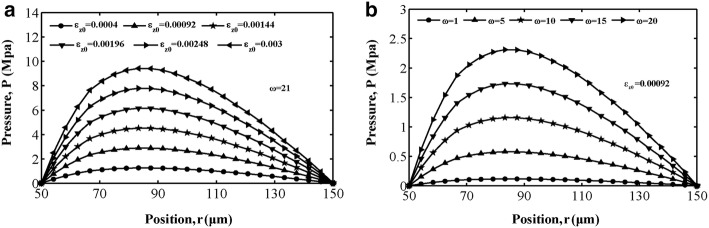
Fluid pressure distribution as a function of the radial displacement *r* for case 2: **a** pressure versus with different strain amplitude at loading frequency $\omega = 21$, **b** pressure versus with different loading frequency at strain amplitude $\varepsilon_{z0} = 0. 0 0 0 9 2$

**Fig. 5:**
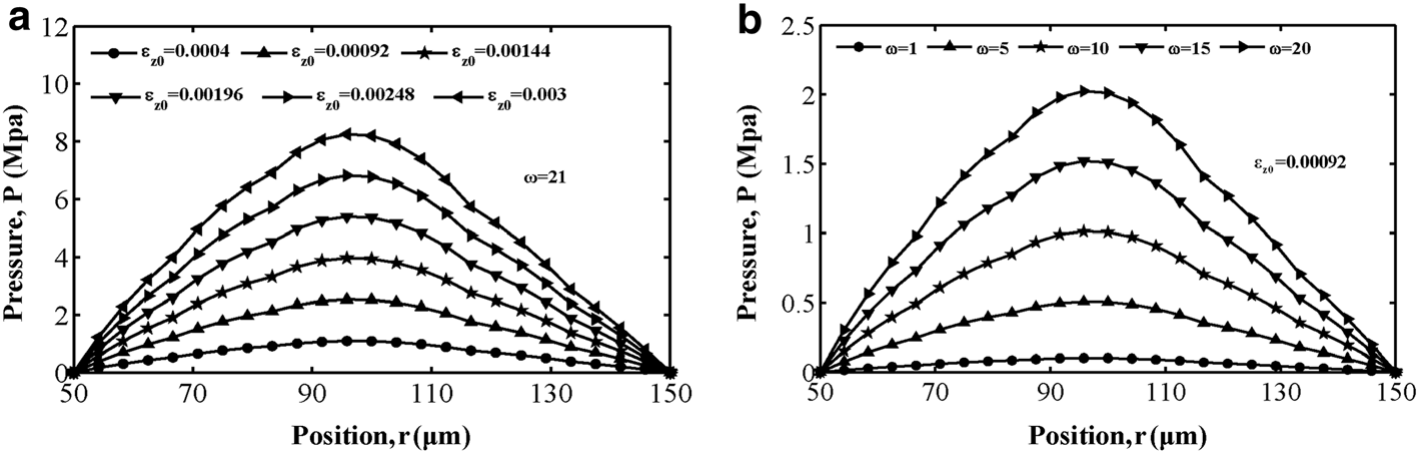
Fluid pressure distribution along the radial displacement *r* in case 3: **a** different strain amplitude at frequency of loading $\omega = 21$ and **b** different loading frequency at strain amplitude $\varepsilon_{z0} = 0. 0 0 0 9 2$

Figures 3a, 4a and 5a show the variations in interstitial fluid pressure distribution for different values of strain amplitude as a function of position for the loading frequencies in cases 1, 2 and 3. We can conclude that at this loading frequency, interstitial fluid pressure varies in amplitude mostly for different strain amplitudes, and the larger the value of the strain amplitude is, the stronger the attractive effect of strain amplitude on the interstitial fluid pressure in all three cases. For cases 1 and 3, the results exhibit an asymmetrical interstitial fluid pressure distribution about the central axis at *r *= 100 μm, owing to the asymmetrical spatial gradient distribution of permeability. For case 2, the spatial gradient of the interstitial fluid pressure in the vicinity of the Haversian canal wall is larger than that around the cement line surface, corresponding to the spatial distribution of permeability. The variation in interstitial fluid pressure *p* with respect to location *r* is depicted in Figs. 3b, 4b and 5b with different values of loading frequency $\omega$ for $\varepsilon_{z0} = 0. 0 0 0 9 2$. In all three figures, increasing the loading frequency $\omega$ caused the interstitial fluid pressure to become larger. As shown in Figs. 3, 4 and 5, interstitial fluid pressure amplitudes in the osteon depend more on the strain amplitude than the loading frequency in a physiological loading state. Thus, strain amplitude plays a key role in governing poroelastic behaviour.

### Effect of strain amplitude on seepage velocity

**Fig. 6:**
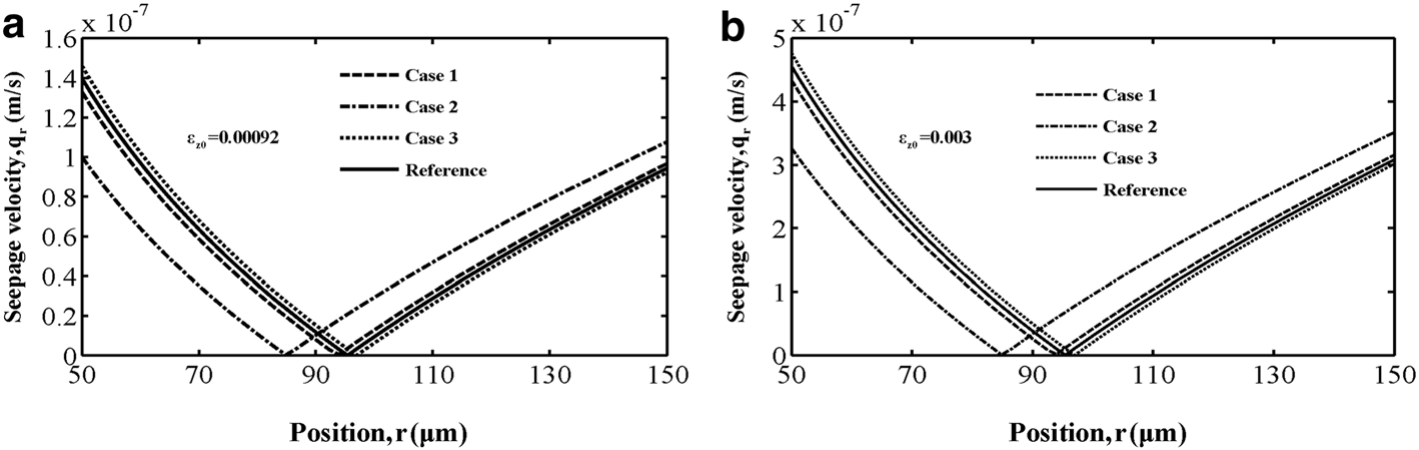
Seepage velocity distribution along the *r*-direction for **a** longitudinal strain amplitude fixed at $\varepsilon_{z0} = 0.00092$ and **b** longitudinal strain amplitude fixed at $\varepsilon_{z0} = 0.003$ in all three cases with the permeability constant $k_{i} = 1.0 \times 1 0^{ - 18} \;(i = 1,\;2, \ldots ,6)$ as reference

In bone materials, the seepage velocity is closely associated with the mechanical stimuli to osteocytes during the bone remodelling process. We studied the seepage velocity distribution on strain amplitude $\varepsilon_{z0}$ inside the single-osteon model. Figure 6 shows the seepage velocity distribution along the *r*-direction in cases 1–3 at the interstitial fluid pressure peak. Figure 6a corresponds to the result for loading strain amplitude $\varepsilon_{z0} = 0.00092$, and Fig. 6b corresponds to the result for $\varepsilon_{z0} = 0.003$. In the two parts of the figures, the results of the seepage velocity in a single osteon with constant permeability, i.e., $k_{i} = 1.0\;(i = 1,2, \ldots ,6)$, are taken as a reference. As shown in Fig. 6a and b, the profiles of the two velocities are very similar, but the seepage velocity value for b is much larger than that for a, which this signifies that the seepage velocity value is related to strain amplitude mostly. We also found that the permeability distribution in each lamella of the osteon is an especially important factor that influences seepage velocity. The maximum value of seepage velocity appears at both ends of the osteon in all the three cases and the reference.

## Discussion

Considering a poroelastic hollow multi-layered slab model subjected to cyclic loading in the longitudinal direction allowed us to study the effect of lamellar structure characteristics and material properties on interstitial fluid pressure and seepage velocity distribution stimuli to osteocytes embedded in an osteon.

The results in this paper have shown that spatial permeability can cause a remarkable variation in interstitial fluid and seepage velocity distributions in the osteon. To understand how bone permeability affects cellular activities in a remodelling bone, Rémond et al. [7] built a finite element model to explain the effect of spatial gradients of permeability on interstitial fluid flow in cortical bone. They pointed out that spatial gradients of permeability do not cause an obvious variation in radial fluid velocity distribution. This is opposite to our results, shown in Fig. 6. However, their results on the effect of the spatial distribution of permeability on interstitial fluid pressure are almost in exact accordance with our work. Such different results may be a consequence of the boundary conditions. Rémond et al. assumed that permeability decreases in a linear fashion from the inside to the outside radius, while ours assumed that different layers between each other have different values of permeability. All lamellae are perfectly bonded to between each other, and the same layer is assumed to be a homogeneous and transversely isotropic material. Wu et al. [1] showed that the velocity changes litter when permeability exceeds 10^−21^ m^2^, and Gatti et al. [10] found that the effect of permeability on velocity is limited when the permeability is larger than 10^−20^ m^2^. The above two references assumed that osteon is homogeneous and listed the permeability span from several orders of magnitude (from 10^−23^ to 10^−21^ m^2^). But in our study, the permeability in different layer was set to be on the same order of magnitude, 10^−18^ m^2^ to suit the continuum mechanics as much as possible. Therefore, fluid velocity is index of interstitial fluid flow stimuli given to osteocytes. Setting an appropriate value of permeability according to the experimental findings can help us better understand the load induced fluid stimuli on osteocytes buried in the lacunar–canalicular system.

Seepage velocity is considered one of the characteristics of mechanical stimuli to osteocytes embedded in lacunar–canalicular poro. Strain amplitude and loading frequency influence the behaviour of the interstitial fluid. Figure 6 shows that when the strain amplitude increases, the seepage velocity and thus the mechanical stimuli to osteocytes are close to both ends of the osteon, while the flow around the centre of the osteon’s lamellae decreases in all three cases. This study implies that osteocytes buried near both ends of the osteon mainly function as mechanosensory cells during the bone remodelling process.

There are several limitations related to the assumptions of this model. First, our attention in this paper was only focused on the contribution of lacunar–canalicular porosity (PLC) on interstitial fluid transport and the mechanotransduction phenomenon in osteons and neglected vascular porosity (PV) and collagen–apatite porosity (PCA). However, the results of Cowin’s [21] study suggest that interstitial fluid pressure in the vascular pores and collagen–apatite pores is lowest compared to pressure in the lacunar–canalicular pores. Therefore, the assumption of only lacunar–canalicular permeability here seems justified. Second, among the material constants listed in Table 1, permeability is the factor that influences interstitial fluid pressure and seepage velocity more remarkably than the others [2]. We selected only permeability because it is considered to be spatially distributed in this model. Finally, we chose the low value of 10^−18^ m^2^ for the PLC as a representative permeability to suit the continuum mechanics [1]. However, setting the appropriate distribution of permeability can help us to understand the mechanisms of physiological activity-induced bone remodelling. This is based on experimental findings. A more sophisticated correlation with the PV, PCA and other material constants would be necessary to address this issue in the future and may contribute to a better understanding of the mechanism of mechanotransduction and the interstitial fluid effects on the bone remodelling process.

## Conclusions

In summary, this analysis provides the distributions of interstitial fluid pressure and seepage velocity in cortical bone and emphasizes the importance of lamellar structure characteristics and material properties, such as permeability. Our results suggest that the amplitude strain of cyclic loading affects pore pressure and fluid velocity remarkably more than loading frequency and interstitial fluid pressure is greatly influenced by permeability variations.

## Abbreviations

PLC: lacunar–canalicular porosity
PV: vascular porosity
PCA: collagen–apatite porosity
